# Supplementary material for: The genome sequence of the fin whale, Balaenoptera physalus (Linnaeus, 1758) (Artiodactyla: Balaenopteridae)
Source: Wellcome Open Res. 2026 Jan 5;11:5. [Version 1] doi: 10.12688/wellcomeopenres.25412.1 (PMC12877476; doi:10.12688/wellcomeopenres.25412.1)
Supplement: Peer review history

# Reviewer report: Reviewer response for version 1

**DOI:** 10.21956/wellcomeopenres.27998.r144117

**Published:** 2026-02-05

**Authors:** L Filipe C Castro

**Affiliations:** 1 University of Porto, Porto, Portugal

Great dataset for a key cetacea species. The methods and description are mostly appropriate.

 minor details missing:

 "The sex chromosomes were identified by the depth of PacBio read coverage." - perhaps some more detail for validation?

 "The genome will be annotated using available RNA-Seq data" - not clear what RNA seq data is (will be) available?

Are sufficient details of methods and materials provided to allow replication by others?

Partly

Is the rationale for creating the dataset(s) clearly described?

Yes

Are the datasets clearly presented in a useable and accessible format?

Yes

Are the protocols appropriate and is the work technically sound?

Yes

Reviewer Expertise:

Comparative Genomics, Evolution and Physiology

I confirm that I have read this submission and believe that I have an appropriate level of expertise to confirm that it is of an acceptable scientific standard.

# Reviewer report: Reviewer response for version 1

**DOI:** 10.21956/wellcomeopenres.27998.r144114

**Published:** 2026-01-24

**Authors:** Mariana F Nery

**Affiliations:** 1 State University of Campinas, São Paulo, Brazil

Great to know that the fin whale genome is ready. This manuscript presents a high-quality, chromosomal-level genome assembly for *Balaenoptera physalus* (fin whale) as part of the Darwin Tree of Life Project. The work is technically sound, and the assembly meets most standard quality benchmarks.

 My only concern relates to sex chromosome identification. The Methods state that sex chromosomes were identified “by the depth of PacBio read coverage,” but the specific criteria used to distinguish them are not described. It is unclear, for example, what coverage thresholds or comparative expectations (e.g., autosomes vs. sex chromosomes) were applied. In addition, I do not readily observe coverage differences in the Hi-C contact map, although I acknowledge that this may reflect my limited expertise with Hi-C–based sex chromosome inference.

Are sufficient details of methods and materials provided to allow replication by others?

Yes

Is the rationale for creating the dataset(s) clearly described?

Yes

Are the datasets clearly presented in a useable and accessible format?

Yes

Are the protocols appropriate and is the work technically sound?

Yes

Reviewer Expertise:

Genomics

I confirm that I have read this submission and believe that I have an appropriate level of expertise to confirm that it is of an acceptable scientific standard.
